# Effects of Pulsed Electric Field Processing and Sous Vide Cooking on Muscle Structure and In Vitro Protein Digestibility of Beef Brisket

**DOI:** 10.3390/foods10030512

**Published:** 2021-03-01

**Authors:** Feng Ming Chian, Lovedeep Kaur, Indrawati Oey, Thierry Astruc, Suzanne Hodgkinson, Mike Boland

**Affiliations:** 1 Riddet Institute, Massey University, 4442 Palmerston North, New Zealand; f.chian@massey.ac.nz (F.M.C.); indrawati.oey@otago.ac.nz (I.O.); S.M.Hodgkinson@massey.ac.nz (S.H.); M.Boland@massey.ac.nz (M.B.); 2 School of Food and Advanced Technology, Massey University, 4442 Palmerston North, New Zealand; 3 Department of Food Science, University of Otago, P.O. Box 56, 9054 Dunedin, New Zealand; 4 INRAE, QuaPA, F-63122 Saint-Genès-Champanelle, France; thierry.astruc@inrae.fr

**Keywords:** pulsed electric field, sous vide cooking, meat structure, in vitro protein digestion

## Abstract

Pulsed electric fields (PEF) in conjunction with sous vide (SV) cooking has been explored for meat tenderisation. The aim of this experiment was to study the effect of PEF–SV treatment on the muscle structure and in vitro protein digestibility of beef brisket. Pulsed electric field treatment (specific energy of 99 ± 5 kJ/kg) was applied to bovine *Deep* and *Superficial pectoral* muscles in combination with sous vide (SV) cooking (60 °C for 24 h). A similar micro- and ultrastructure was detected between the control SV-cooked and PEF-treated SV-cooked *pectoral* muscles. The combined PEF–SV treatment increased the in vitro protein digestibility of the *pectoral* muscles by approximately 29%, in terms of ninhydrin-reactive free amino nitrogen released at the end of simulated digestion. An increment in proteolysis of the PEF-treated SV-cooked meat proteins (e.g., myosin heavy chains and C-protein) during simulated digestion was also observed using sodium dodecyl sulfate-polyacrylamide gel electrophoresis. More damaged muscle micro- and ultrastructure was detected in PEF-treated SV-cooked muscles at the end of in vitro digestion, showing its enhanced digestive proteolysis compared to the control cooked meat.

## 1. Introduction

Pulsed electric field (PEF) is a food processing technique that applies short electric pulses to food products, leading to the electroporation of the cell membrane when the induced transmembrane potential exceeds a critical value of 1 volt [1]. In recent years, low-intensity pulsed electric field processing, alone or in combination with other processing techniques, has been explored for meat tenderisation. The combined PEF–sous vide (SV) cooking process [2], the combined freezing–PEF process [3], and the combined PEF–aging process [4,5] have been reported to promote meat tenderisation. For instance, Alahakoon et al. [2,6] reported that a PEF treatment at 1.5 kV cm^−1^ in conjunction with sous vide cooking at 60 °C for 20.8 to 23.7 h improved the tenderness of beef briskets. The shear force of the frozen–thawed beef *Semitendinosus* muscles treated with PEF at 1.4 kV/cm, 50 Hz, and 20 μs was also reduced by 20% [3].

As consumers usually seek food products that are tasty and nutritious, it is important to understand the effects of the different food processing combinations, such as the combined PEF–SV process, on the nutritional value of meat products, for example the protein digestibility [7]. Current research performed to understand the effects of PEF in combination with cooking on meat protein digestibility is limited, particularly in regard to understanding of the structural changes that occur during digestion. The in vitro protein digestibility of PEF-treated water bath-cooked (core temperature of 75 °C) bovine *Semimembranosus* muscles has been reported to be higher than that of the control untreated cooked meat [8]. Conversely, Alahakoon et al. [4] did not detect any effect of the combined PEF (0.7 kV/cm, 90 to 100 kJ/kg)–SV (60 °C for 24 h) process on the in vitro protein digestibility of beef brisket. The impact of the structural changes induced by a combined PEF–cooking process on the enzymatic breakdown of meat proteins during digestion has not been explored and requires further research.

Thus, this research aims to examine the in vitro protein digestibility of PEF-treated SV-cooked beef brisket using both biochemical and microscopy approaches. As both low-intensity PEF treatment [9,10] and cooking [11,12] have been reported to induce structural changes in muscle-based foods, it was hypothesised that the combined PEF–SV cooking treatment would modify meat structure and affect the degradation of muscle protein during subsequent simulated digestion, leading to the improved protein digestive properties of the meat.

## 2. Materials and Methods

### 2.1. Pulsed Electric Field Processing and Sous Vide Cooking of Beef Brisket

**Figure 1:**
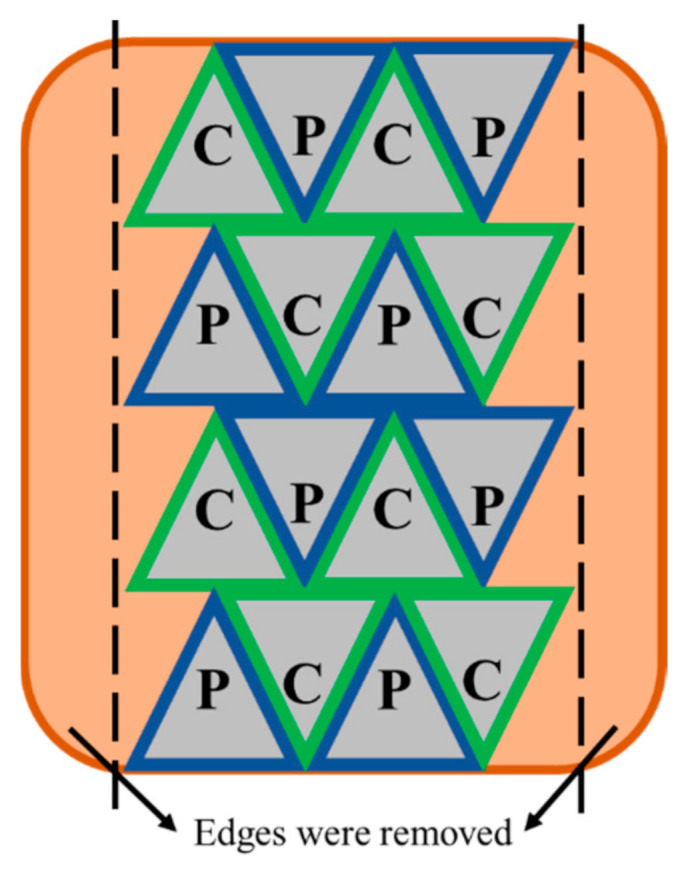
Meat sampling position of the control untreated (C) and the pulsed electric field (PEF)-treated (P) samples on the whole brisket. The edges of the brisket which were too thin for PEF processing were removed. In order to minimise the inconsistency due to muscle inhomogeneity, sampling was performed by allocating the C and P adjacent to each other. The meat was cut into triangular pieces of about 70 g with dimensions of 6 cm in height, 4 cm in width and 6 cm in length for PEF treatment.

Whole beef briskets (*Deep* and *Superficial pectoral* muscle) from a Hereford sired heifer (19 months old, mix of Friesian and crossbreed, 195.5–270.0 kg carcass weight) were obtained from ANZCO Foods (Eltham, New Zealand). The pre-rigor brisket was stored at 15 °C for 48 h until it went into rigor. The brisket was then vacuum-packed, blast-frozen, and kept at −18 °C until PEF treatment. The meat was thawed at 4 °C for 18 h before PEF treatment. The pulsed electric field treatment was conducted in a pilot-scale batch PEF system (Elcrack-HVP 5, DIL, Quakenbruck, Germany). After removing the edges of the whole brisket which were too thin for the treatment, the meat was cut into triangular shapes that were 6 cm in height, 4 cm in width and 6 cm in length, with an approximate weight of 70 g (Figure 1). The PEF–SV process was carried out based on the processing parameters optimised by Alahakoon et al. [2,6], whereby the PEF-treated SV-cooked beef briskets have the optimal tenderness and quality (e.g., colour and cook loss).

In brief, the PEF treatment was carried out using processing parameters of 0.7 kV/cm electric field strength to achieve a specific energy of 99 ± 5 kJ/kg, at a constant pulse width and frequency of 20 µs and 50 Hz, respectively. The specific energy was determined using Equation (1).

Specific energy input (kJ/kg) = pulse number × pulse energy/sample weight(1)

The conductivity of the brisket was measured at more than 25 different positions across the whole brisket, using a handheld meat conductometer (LF-STAR, R. Mathäus, Nobitz, Germany), before and after PEF treatment. The pre- and post-PEF treatment conductivities of the meat were 9 ± 2 mS/cm and 13 ± 1 mS/cm, respectively. The temperature of the meat had increased by about 14.0 °C after PEF treatment to 22.4 °C.

After PEF treatment, both control and PEF-treated meat samples were vacuum-packed in “cook-in” clear vacuum bags (Cas-Pak Products Ltd., Silverdale, New Zealand). The vacuum-packed samples were then SV-cooked at 60 °C for 24 h. The cooked samples were snap-frozen using liquid nitrogen and afterwards stored at −80 °C for further analyses.

### 2.2. In Vitro Protein Digestibility

#### 2.2.1. In Vitro Digestion

The in vitro digestion of the SV-cooked meat was conducted as described by Chian et al. [10] with modifications. The information of the digestive enzyme types and concentrations, as well as the sampling time points in each digestion phase (oral, gastric and small intestinal phase) is tabulated in Table 1. Glass balls (3–5 mm) were added to the digestion reactors to mimic sample maceration. The digests (20 mL) were sampled at 0, 30 and 60 min of gastric digestion (cumulative digestion times of 2, 32 and 62 min) and 60 and 120 min of small intestinal digestion (cumulative digestion time of 122 and 182 min). After sampling, 12 µL of pepstatin A (ab141416, Abcam, Cambridge, UK) (0.5 mg/mL in methanol) was added immediately to every mL of gastric digest to inactivate pepsin, while 0.45 mL of SIGMAFAST^™^ protease cocktail solution (S8820, Sigma-Aldrich, Saint Louis, MO, USA) (1 tablet/50 mL MilliQ water) was added to every mL of intestinal digest to inactivate pancreatic proteases, before storage at −20 °C [13]. The digests were processed as described by Chian et al. [10] for subsequent analyses of tricine sodium dodecyl sulfate–polyacrylamide gel electrophoresis (SDS-PAGE) and ninhydrin-reactive free amino nitrogen.

#### 2.2.2. Sodium Dodecyl Sulfate-Polyacrylamide Gel Electrophoresis (SDS-PAGE) and Ninhydrin-Reactive Amino Nitrogen Analysis

Reducing tricine–SDS-PAGE was performed on a 16.5% Criterion^™^ Tris-tricine gel (3450064, Bio-Rad Laboratories, Hercules, CA, USA) [10]. Each lane was loaded with 20 µg of protein after adjusting the protein concentration of the digests–tricine sample buffer mixture (1610739, Bio-Rad Laboratories, Hercules, CA, USA) (1:1 ratio), based on the nitrogen concentration of the digests determined using the Kjeldahl method [14]. The electrophoresis was then conducted with a Criterion^™^ Vertical Electrophoresis system (1656001, Bio-Rad Laboratories, Hercules, CA, USA) at a constant voltage of 125 V until the tracking dye front reached the end of the gel. The ninhydrin-reactive free amino nitrogen released at different digestion time points was determined using ninhydrin reagent (N7285, Sigma-Aldrich, Saint Louis, MO, USA) [15]. The amount of ninhydrin-reactive free amino nitrogen released at different digestion time points was calculated using Equation (2).

Ninhydrin-reactive amino nitrogen released (%) = Ninhydrin-reactive amino nitrogen in the digests ÷ total nitrogen present in meat × 100(2)

### 2.3. Microscopy Analysis of Muscles

The control SV-cooked and the PEF-treated SV-cooked samples were subjected to simulated oral–gastro–small intestinal digestion, as described in Section 2.2.1 in a polyester mesh without the use of glass balls. Samplings were conducted at different in vitro digestion time points for histochemical (0 min, 62 min and 182 min) and transmission electron microscopy (TEM) (0 min and 182 min) analyses. The samples for histochemical analysis were cut in half by length and cryofixed in liquid nitrogen (−196 °C)-cooled isopentane (−160 °C) for at least 30 s, and were stored at −80 °C. The samples for TEM analysis were cut along the muscle fibre into strips (10 mm × 3 mm × 3 mm) and were fixed in 2.5% glutaraldehyde in 0.1 M sodium cacodylate buffer at pH 5.6 overnight at room temperature before storage at 4 °C.

#### 2.3.1. Histochemical Analysis

The cryofixed muscle blocks were cut into 10 µm thick sections (cross-section) using a cryostat (CM1950, Leica Microsystems GmbH, Wetzlar, Germany) at −20 °C. The sections were mounted on glass slides and air-dried for at least an hour and subsequently stained with Picro-Sirius Red [16]. In brief, the muscle sections were incubated in acetone for an hour followed by picroformalin (5% formaldehyde and 90% ethanol in saturated picric acid) for 10 min. Then, the sections were submerged in 90% ethanol for 1 min, distilled water for 10 min and Picro-Sirius Red stain (0.1% Sirius Red in saturated aqueous picric acid) for 1 h. After that, the sections were put in a bath of 0.01 M hydrochloric acid for 5 min and distilled water for 1 min. Finally, the sections were dehydrated by dipping the slides into a bath of 95% ethanol and two baths of 100% ethanol, followed by two baths of methycyclohexane. The microstructure was observed using an optical transmission microscope coupled to a digital acquisition kit (Olympus BX61 microscope, Olympus DP 71 digital camera, Olympus France SAS, Rungis, France).

#### 2.3.2. Transmission Electron Microscopy (TEM) Analysis

The chemically fixed samples were post-fixed in 1% osmium tetroxide in 0.1 M sodium cacodylate buffer (pH 7.2) and were dehydrated through a graded series of ethanol (70%, 95% and 100%) [17]. The dehydrated sections were then embedded in epoxy resin (TAAB, Eurobio Scientific, Les Ulis, France), followed by sectioning into 90 nm of ultra-thin sections. The ultra-thin sections were mounted on copper grids, followed by staining with saturated uranyl acetate and lead citrate (30 min each) [18]. Observations were carried out using a transmission electron microscope (HM 7650, Hitachi, Tokyo, Japan) coupled with a charge-coupled device (CCD) Advanced Microscopy Techniques (AMT) high resolution (HR) digital camera system (Hamamatsu Photonics, Shizuoka, Japan). The reagents used in this section were electron microscopy grade (Electron Microscopy Science, Hatfield, PA, USA).

### 2.4. Statistical Analysis

The data were reported as means ± standard deviation of means from four replications. Repeated measures analysis of variance (ANOVA) by Generalized Linear Model (IBM^®^ SPSS^®^ Statistic version 25, IBM Corporation, Armonk, NY, USA) was performed for protein digestibility analysis, followed by Tukey’s post-hoc analysis to assess the significance of difference at a confidence level of 0.05. No violation of sphericity was detected using Mauchly’s Test.

## 3. Results and Discussion

### 3.1. Protein Digestibility

#### 3.1.1. Sodium Dodecyl Sulfate–Polyacrylamide Gel Electrophoresis (SDS-PAGE) Analysis

**Figure 2:**
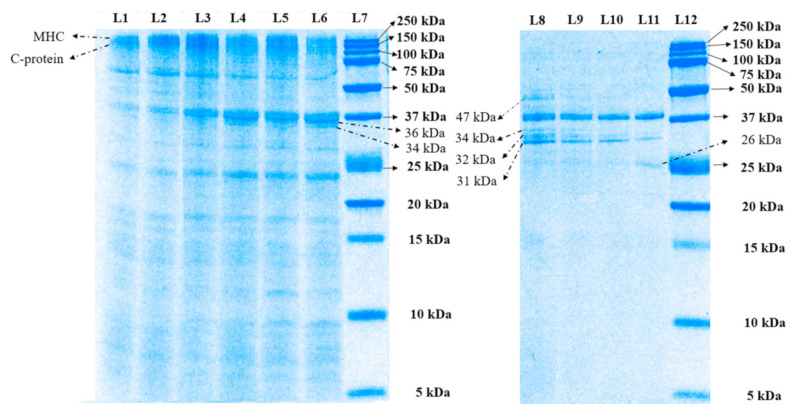
Tricine SDS-PAGE electrophoretogram displaying the protein profile of the digests of control sous vide (SV)-cooked and pulsed electric field (PEF)-treated SV-cooked meat during simulated digestion. L7 and L12 are the molecular weight standard, labelled in kDa. L1, L3 and L5 denote control SV-cooked samples at 2, 32 and 62 min of oral–gastric digestion, respectively. L2, L4 and L6 denote PEF-treated SV-cooked samples at 2, 32 and 62 min of oral–gastric digestion, respectively. L8 and L10 represent control SV-cooked samples at 122 and 182 min of oral–gastric–small intestinal digestion, respectively. L9 and L11 represent PEF-treated SV-cooked samples at 122 and 182 min of oral–gastric–small intestinal digestion, respectively. The protein bands were identified on the electrophoretogram as described by Kaur et al. [19,24] and Boland et al. [20]. MHC stands for myosin heavy chain.

The proteolysis of the meat proteins during in vitro oral–gastro–small intestinal digestion was studied using tricine SDS-PAGE (Figure 2). During 62 min of in vitro oral–gastric digestion, some differences in the intensities of the protein bands were observed. After 32 min of simulated oral–gastric digestion, the band intensities of the myosin heavy chain (MHC, 220 kDa) and C-protein (140 kDa) of the digest of the PEF-treated SV-cooked muscles were lighter than the digest of the control SV-cooked muscles, indicating more breakdown of these proteins in the former [19,20]. In addition, the intensity of the band with molecular weight 36 kDa of the digest of PEF-treated SV-cooked muscles was higher than the control untreated SV-cooked meat digest. A new band with molecular weight 34 kDa was observed in the digest of the treated sample only. Protein bands with molecular weights of 36 kDa and 34 kDa have been reported to be the α- and β-subunit of the β-actinin, respectively [21]. Similar observations were also made at the end of the simulated oral–gastric digestion (62 min), with the PEF-treated SV-cooked samples displaying increased gastric proteolysis compared to the control SV-cooked samples.

After 122 min of in vitro oral–gastro–small intestinal digestion, high molecular-weight proteins (molecular weight > 50 kDa) of both control SV-cooked and PEF-treated SV-cooked samples were fully digested by the action of pancreatin. A protein band with a molecular weight of 47 kDa was found in the digest of control SV-cooked meat but not in the digest of the PEF-treated SV-cooked meat. This band has previously been identified as β-enolase [22] or the degradation product of desmin during the aging process [23]. An additional hour of digestion resulted in the complete disappearance of this band in the digest of the control SV-cooked meat. This observation shows that the protein corresponding to this band (47 kDa) was digested faster in the PEF-treated SV-cooked meat. Moreover, the bands with molecular weights of 34 kDa, 32 kDa, and 31 kDa were found in both samples at the end of 122 min of simulated digestion, with the intensities of these bands higher in the control SV-cooked meat digests. The protein bands with molecular weights of 34 kDa and 32 kDa (unidentified protein) were not detected in the digest of both the control and the treated samples at the end of 182 min of simulated digestion. At the same time, the intensity of the band with a molecular weight of 31 kDa (unidentified protein) was further reduced, with a higher band intensity found in the digest of the control SV-cooked samples. Furthermore, a new band with a molecular weight of 26 kDa, which could be the hydrolysis product of higher molecular weight proteins, formed only in the digest of PEF-treated SV-cooked meat [19]. These observations demonstrated that the PEF-treated SV-cooked meat was better hydrolysed when compared to the untreated SV-cooked meat during simulated digestion. Bhat et al. [8] also observed a greater and faster proteolysis of PEF-treated cooked beef. He proposed that the PEF treatment resulted in protein structural changes and improved membrane permeability in meat, increasing the availability of proteolytic sites to digestive enzymes. Overall, the SDS-PAGE analysis demonstrated that PEF treatment influenced the digestive properties of the SV-cooked meat, whereby the protein and peptide profile of the meat digests was modified.

#### 3.1.2. Ninhydrin-Reactive Amino Nitrogen Analysis

The ninhydrin-reactive amino nitrogen released by meat samples at different digestion time points was determined as a quantitative measure of in vitro protein digestibility. A higher percentage of ninhydrin-reactive amino nitrogen indicates a greater extent of protein hydrolysis by the digestive enzymes. As summarised in Table 2, there was no difference in the percentage of ninhydrin-reactive amino nitrogen released after 122 min of in vitro oral–gastro–small intestinal digestion between the control SV-cooked and the PEF-treated SV-cooked meat (*p* > 0.05). However, at the end of 182 min of simulated digestion, there was significantly more ninhydrin-reactive amino nitrogen released from the PEF-treated SV-cooked meat than from the control SV-cooked meat. Overall, the combined PEF–SV process increased the in vitro oral–gastro–small intestinal protein digestibility by 28.6%, which is in agreement with the findings of an increased proteolysis of the PEF-treated SV-cooked meat as discussed in Section 3.1.1. However, Alahakoon et al. [4] reported that the in vitro protein digestibility of beef brisket was unaffected by the combined PEF–SV cooking process. Although the experiment was conducted with the same meat cut (beef brisket) and processing parameters (0.7 kV/cm, 90 to 100 kJ/kg), the authors reported no significant difference in the trichloroacetic acid (TCA)-soluble peptides concentration between the control SV-cooked and PEF-treated SV-cooked meats at the end of the simulated digestion. This might be due to the difference in the assay used in determining the extent of proteolysis. Measuring the degree of hydrolysis with TCA-soluble peptide concentrations assumes that all the intact proteins are precipitated by TCA, and only small peptides and amino acids remain in the soluble fraction [25]. However, this assumption might be incorrect as TCA has been reported to be unable to precipitate up to 70% of proteins or peptides larger than 10 kDa from ileal digest. Thus, quantifying TCA-soluble peptides could overestimate the degree of proteolysis if the digest contains more of the larger peptides that cannot be precipitated by TCA. In addition, this method does not quantify the cleavage of peptide bonds [25]. Pulsed electric field treatment (0.6 kV/cm, 73.28 kJ/kg) was found to improve the in vitro protein digestibility of water bath-cooked bovine *Semitendinosus* (core temperature of 75 °C) by approximately 2%, based on the protein content left undigested in the samples, quantified using the Kjeldahl method [8].

### 3.2. Structural Changes of Meat Samples during Simulated Digestion

#### 3.2.1. Microstructure Studied Using Histochemical Analysis

**Figure 3:**
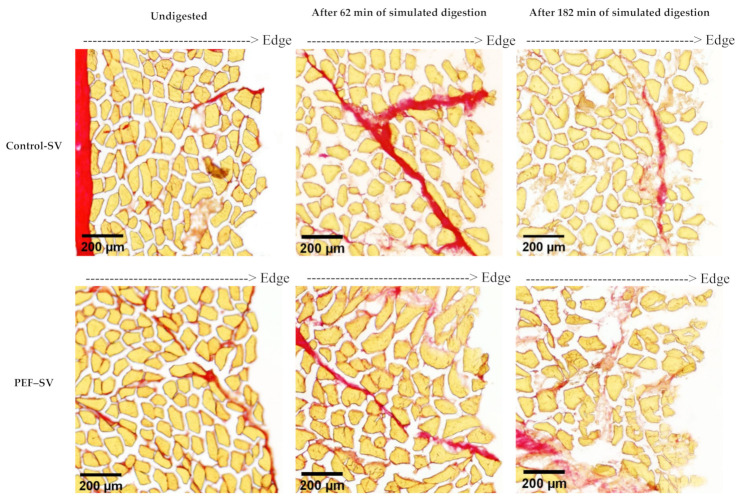
Histological sections of the control sous vide (SV)-cooked and the pulsed electric field (PEF)-treated SV-cooked meat at different digestion time points, showing more severe structural degradation of PEF-treated meat by the digestive enzymes at the end of simulated digestion. Connective tissue (in red) was stained with Sirius Red dye and muscle cells (in yellow) were stained with picric acid.

The microstructure of the control SV-cooked and the PEF-treated SV-cooked meat at different stages of in vitro digestion is shown in Figure 3. Pulsed electric field treatment did not affect the microstructure of the SV-cooked meat. The microstructure of both control and PEF-treated samples appeared to be similar after SV cooking. Although a reduction in muscle cell sizes and the formation of gaps between muscle fibres were observed in PEF-treated raw muscles by Gudmundsson and Hafsteinsson [9], these observations were not detected in PEF-treated SV-cooked muscles in this experiment. This might be due to the effect of SV cooking on muscle structure being larger and thus masking the effect of PEF on the muscle microstructure. After 62 min of in vitro oral–gastric digestion, the muscle structures of both the control SV-cooked and PEF-treated SV-cooked muscles were damaged at the edges of the meat sections. Further in vitro digestion with the addition of pancreatin at pH 7.0 ± 0.1 for 2 h resulted in more breakdown of the muscle cells and connective tissues, while the disruption extended towards the core of the samples. The damage to the muscle structure was greater in the PEF-treated SV-cooked samples at the end of both in vitro oral–gastric and oral–gastro–small intestinal digestion. This indicates that the PEF-treated SV-cooked brisket was more susceptible to enzymatic hydrolysis by the digestive enzymes, which is in agreement with the outcomes from the SDS-PAGE and ninhydrin-reactive amino nitrogen analyses reported in Section 3.1.1 and Section 3.1.2, respectively. Swollen muscle cells were observed in both control SV-cooked and PEF-treated SV-cooked meat after 62 min of simulated oral–gastric digestion, with an enhanced swelling effect observed in the PEF-treated meat. Bordoni et al. [26] observed the swelling phenomena of muscle cells due to the penetration of the saliva and gastric juices into the meat matrix during simulated digestion. The swelling of muscle cells during gastric digestion has been reported to be due to the effect of acidic gastric juice, but not the action of pepsin [27]. Astruc detected an increment in the muscle cell size in cooked meat samples incubated in simulated gastric juice without the addition of pepsin. Acidic pH (pH < 3.52) resulted in an increment in the net positive charges on the myofibrillar proteins, which increased the electrostatic repulsion forces between protein molecules [28]. This might result in larger spaces between the myofilaments, allowing the penetration of the digestive juice into the meat matrix. In addition, PEF processing has been reported to lead to the formation of pores in cell membranes due to electroporation, enhancing the mass transport and diffusion process in meat [29]. Thus, the diffusion of digestive juices into the meat matrix might be improved by PEF processing. Future studies should be carried out to examine the cell membrane structure of meat to validate this suggestion. The enhanced penetration of digestive juices facilitates the accessibility of digestive enzymes to their substrates, enabling enzymatic protein hydrolysis. Thus, the increased enzymatic breakdown of PEF-treated SV-cooked muscle structure could be a consequence of the enhanced diffusion of digestive juices, displaying as more swollen cells of the PEF-treated samples during simulated digestion, as observed in Figure 3, promoting the action of digestive enzymes.

#### 3.2.2. Ultrastructure Studied Using TEM Analysis

**Figure 4:**
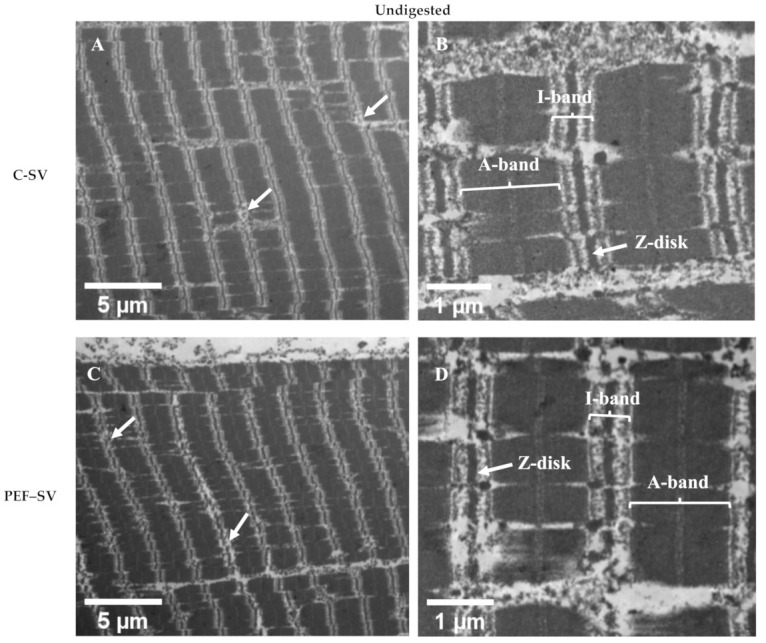
Transmission electron micrographs showing the ultrastructure of the control (**C**) sous vide (SV)-cooked (**A**,**B**) and the PEF-treated SV-cooked (**C**,**D**) beef brisket before simulated oral–gastro–small intestinal digestion.

As presented in Figure 4, the ultrastructure of the control SV-cooked and PEF-treated SV-cooked meats was similar. Pulsed electric field treatment did not affect the ultrastructure of the SV-cooked meat. Ultrastructural modification has been reported in PEF-treated raw muscle (without SV). Elongated sarcomeres [10] and sarcomeres with jagged edges [3] were found in low-intensity PEF-treated uncooked meat. However, these were not observed in the PEF-treated SV-cooked meat. In contrast, both control SV-cooked and PEF-treated SV-cooked meats had coagulated myofibrils accompanied by the formation of granular aggregates, which have previously been detected in thermally treated muscles [17,30,31]. These observations show that SV cooking had a major effect on the muscle ultrastructure when compared to PEF treatment. In addition, the degradation of the sarcomeres along the I-band and Z-disk junctions was observed in both control and PEF-treated muscles after SV cooking at 60 °C (white arrows in Figure 4A,C). The effect of SV cooking on the degradation of sarcomeres along the I-band and Z-disk junctions is considered minor, as the denaturation temperatures of the major I-band- and Z-disk-associated proteins are mostly higher than 60 °C (SV cooking temperature). For instance, actin, which is the major component of I-bands and the core of a Z-disk, has the maximum thermal denaturation temperature (T_max_) range, from 70 to 80 °C [32,33]. Nevertheless, the prolonged heating of meat might result in a small proportion of actin denaturation at a temperature below its T_max_, but above its denaturation onset temperature [34]. The disintegration of thin filaments at the I-band and Z-disk junctions were observed in bovine muscles cooked to an internal temperature of 63 °C, and the extent of disintegration increased as the final internal temperature raised to 73 °C [31]. The degradation of the sarcomeres along the I-band and Z-disk junctions could also be due to postmortem proteolysis [27] and/or protein hydrolysis during the initial stage of SV cooking by the action of endogenous enzymes. The modification of the I-band and Z-disk junctions has been detected in aged muscles as a result of postmortem proteolysis [27,35]. Endogenous enzymes such as cathepsins, which are relatively more heat stable, are likely to contribute to meat tenderisation during low-temperature, long-time cooking processes [36]. The activities of cathepsin B and L were still measurable in *Semitendinosus* muscles obtained from both cows and young bulls after cooking at 63 °C for 19.5 h [37]. Kaur et al. [38] also reported that cathepsins B and L were inactivated after incubating beef brisket at 60 °C for 5 h. Cathepsins B and L in porcine *Semitendinosus* and *Longissimus* muscles have been reported to stay active at 58 °C for 17 h [39]. As PEF treatment has been proposed to aid in the release of cathepsins from the lysosomes by electroporation, the meat tenderisation process could be enhanced during the initial stage of low-temperature long-time cooking [29,40]. Structure disruption due to postmortem proteolysis and the action of endogenous enzymes during low-temperature long-time cooking might promote the action of digestive enzymes during subsequent digestion [27]. In addition to SV cooking, freezing and thawing processes prior to PEF processing might have altered the ultrastructure of meat, thus masking the effect of PEF on meat structure [41]. Faridnia et al. [3] reported that PEF processing (250 kJ/kg) led to significant ultrastructural changes in meat when compared to the freezing and thawing process. However, as the PEF intensity used in this experiment was milder, it might not have had the same effect as that observed by Faridnia et al. Thus, future studies should be conducted with fresh meat samples to better determine the effect of PEF alone and PEF–SV processing on meat structure.

**Figure 5:**
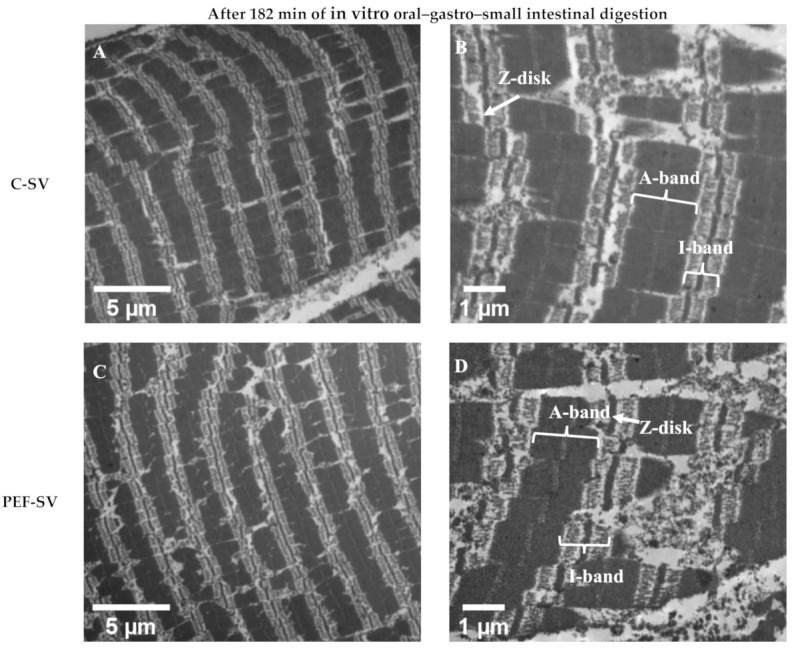
Transmission electron micrographs displaying the ultrastructure of the control (**C**) sous vide (SV)-cooked (**A**,**B**) and the PEF-treated SV-cooked (**C**,**D**) beef brisket after 182 min of simulated digestion. The digested PEF-treated SV-cooked meat had more damaged sarcomeres and more coagulated and elongated I-bands, indicating more severe proteolysis by the digestive enzymes.

After 182 min of in vitro oral–gastro–small intestinal digestion, the breakdown of the myofibrils was observed in both control SV-cooked and PEF-treated SV-cooked muscles (Figure 5). The Z-disks were degraded and the sarcomeres were broken down. The more severe disruption of Z-disks and sarcomeres was observed in the digested PEF-treated SV-cooked muscles than in the digested control SV-cooked muscles. This shows that the PEF-treated SV-cooked muscles were more susceptible to proteolysis by the digestive enzymes, which is consistent with the microstructure analysis as discussed above. In addition, more coagulated and elongated I-bands were found in the digested PEF-treated SV-cooked muscles. This was also observed in the digested raw PEF-treated bovine *Longissimus thoracis* muscles, where the digested PEF-treated raw muscles had better protein digestibility than the untreated samples [10]. The more significantly coagulated I-bands of the digested PEF-treated muscles might be due to the more significant acid denaturation of the protein by the gastric juices, which would have exposed buried peptide bonds, allowing the access of digestive enzymes, leading to increased proteolysis [10,42].

## 4. Conclusions

The in vitro protein digestibility of meat was significantly increased by the combined PEF–SV process, whereby the PEF-treated SV-cooked meat had higher ninhydrin-reactive amino nitrogen (*p* < 0.05) released at the end of the simulated digestion, and increased proteolysis observed using tricine–SDS-PAGE, compared to the control SV-cooked meat. The improvement in protein digestibility might be due to the disruption of muscle structure by the combined PEF–SV process. Although the muscle micro- and ultrastructure of the control SV-cooked and PEF-treated SV-cooked meat was similar, their muscle structures changed differently during simulated digestion. More swollen muscle cells were observed in the PEF-treated SV-cooked meat after 62 min of simulated oral–gastric digestion, suggesting the enhanced penetration of digestive juices in the treated samples. The enhanced penetration of digestive juices is postulated to be due to the formation of pores in muscle cell membranes as a result of the electroporation effect of PEF, facilitating the accessibility of digestive enzymes to their substrates. A more damaged muscle microstructure and ultrastructure was also detected in the PEF-treated SV-cooked muscles at the end of in vitro oral–gastro–small intestinal digestion, compared to the control cooked samples. These observations show that a combination of PEF treatment and SV cooking process affected muscle structural changes during simulated digestion, leading to an improved in vitro protein digestibility of the meat.

## Figures and Tables

**Table 1:** Digestive enzyme types and concentrations, digestion duration and sampling time points for each digestion phase (oral, gastric and small intestinal).

Digestion Phase	Enzyme Types	Enzyme Concentrations	Digestion Duration (min)	Cumulative Digestion Time (min)	Sampling Time (min) Based on Cumulative Digestion Time
Oral (pH 7 ± 0.1)	α-amylase (10025, Sigma Aldrich, Saint Louis, MO, USA)	1.25 × 10^−6^ katal /mL bolus	2	2	No
Gastric (pH 3 ± 0.1)	Pepsin (P7125, Sigma Aldrich, Saint Louis, MO, USA)	1.33 × 10^−7^ katal/mg meat protein	60	62	2, 32, 62
Small intestinal (pH 7 ± 0.1)	Pancreatin (P1750, Sigma Aldrich, Saint Louis, MO, USA)	1:100 pancreatin to meat protein ratio	120	182	122, 182

**Table 2:** Ninhydrin-reactive amino nitrogen released from the control sous vide (SV)-cooked and the pulsed electric field (PEF)-treated SV-cooked meat after in vitro oral–gastric (2, 32 and 62 min) and further small intestinal (122 and 182 min) digestion.

Cumulative Digestion Time (min)		2	32	62	122	182
Ninhydrin-reactive amino nitrogen (%)	Control–SV	1.9 ± 0.0 ^a,A^	2.3 ± 0.4 ^a,A^	2.7 ± 1.0 ^a,A^	8.1 ± 1.2 ^b,A^	9.8 ± 0.6 ^c,A^
	PEF–SV	1.9 ± 0.0 ^a,A^	2.7 ± 0.6 ^a,b,A^	3.4 ± 1.0 ^b,A^	8.4 ± 0.4 ^c,A^	12.6 ± 0.7 ^d,B^

Values with different lower-case letters within the same row differ significantly (*p* < 0.05). Values with different upper-case letters within the same column differ significantly (*p* < 0.05). Data are shown as means ± standard deviation of means. *n* = 4 (four replicates with three measurements for each replicate).

## Data Availability

The data are available on request from the corresponding author (L.K.).
